# Deep learning is a promising technology and seems to be the future of the CT stone evaluation

**DOI:** 10.1590/S1677-5538.IBJU.2022.0132.1

**Published:** 2022-08-03

**Authors:** Alexandre Danilovic

**Affiliations:** 1 Faculdade de Medicina da Universidade de São Paulo Hospital das Clínicas Departamento de Urologia São Paulo SP Brasil Departamento de Urologia, Hospital das Clínicas, Faculdade de Medicina da Universidade de São Paulo - FMUSP, São Paulo, SP, Brasil; 2 Hospital Alemão Oswaldo Cruz São Paulo SP Brasil Hospital Alemão Oswaldo Cruz, São Paulo, SP, Brasil

## COMMENT

Computed tomography (CT) is the current gold standard diagnostic imaging exam for urolithiasis (1). However, making a CT report is a time-consuming process and requires a specialist. Therefore, an automated model of kidney stones detection would help saving health resources.

The authors of “Deep learning model-assisted detection of kidney stones on computed tomography” showed that a convolution-based algorithm, xResNet50, detected kidney stones with accuracy up to 85.0% for 0-1 cm stones, 89.0% for 1-2 cm stones and 93.0% for > 2 cm stones in CT sagittal section compared to experienced radiologists. Not surprisingly, larger stones are easier to detect (1). However, the accuracy of this automated model to detect kidney stones seems to be not sufficient to dismiss the specialist analysis. Although detection of stones is a good primary objective for an automated model, the mere detection of a kidney stone is not enough for clinical application. A complete report of the stone features is necessary for the best clinical decision. Also, the automated model algorithm should take in consideration CT settings as tube current and window as it impacts measurements of clinically relevant stone features such as size and density (2, 3).

However, artificial intelligence is advancing fast. Other authors were able to show good agreement of other automated model algorithm with radiologist results for stone size, volume, location, number and density (4, 5). Deep learning is a promising technology and seems to be the future of the CT stone evaluation.
